# Predicting the body core temperature of recreational athletes at the end of a 10 km self‐paced run under environmental heat stress

**DOI:** 10.1113/EP091017

**Published:** 2023-04-05

**Authors:** Marcelo T. Andrade, Matheus M. S. Nunes‐Leite, Rúbio S. Bruzzi, Carlos H. Souza, João P. Uendeles‐Pinto, Luciano S. Prado, Danusa D. Soares, Dawit A. P. Gonçalves, Cândido C. Coimbra, Samuel P. Wanner

**Affiliations:** ^1^ Exercise Physiology Laboratory, School of Physical Education, Physiotherapy and Occupational Therapy Universidade Federal de Minas Gerais Belo Horizonte MG Brazil; ^2^ Sports Training Center, School of Physical Education, Physiotherapy and Occupational Therapy Universidade Federal de Minas Gerais Belo Horizonte MG Brazil; ^3^ Laboratory of Endocrinology and Metabolism, Institute of Biological Sciences Universidade Federal de Minas Gerais Belo Horizonte MG Brazil

**Keywords:** heat, heart rate, hyperthermia, physical exercise, regression, self‐paced, speed, temperature, thermoregulation, wet‐bulb globe temperature

## Abstract

Measurement of body core temperature (*T*_core_) is paramount to determining the thermoregulatory strain of athletes. However, standard measurement procedures of *T*_core_ are not practical for extended use outside the laboratory environment. Therefore, determining the factors that predict *T*_core_ during a self‐paced run is crucial for creating more effective strategies to minimize the heat‐induced impairment of endurance performance and reduce the occurrence of exertional heatstroke. The aim of this study was to identify the factors predicting *T*_core_ values attained at the end of a 10 km time trial (end‐*T*_core_) under environmental heat stress. Initially, we extracted data obtained from 75 recordings of recreationally trained men and women. Next, we ran hierarchical multiple linear regression analyses to understand the predictive power of the following variables: wet‐bulb globe temperature, average running speed, initial *T*_core_, body mass, differences between *T*_core_ and skin temperature (*T*_skin_), sweat rate, maximal oxygen uptake, heart rate and change in body mass. Our data indicated that *T*_core_ increased continuously during exercise, attaining 39.6 ± 0.5°C (mean ± SD) after 53.9 ± 7.5 min of treadmill running. This end‐*T*_core_ value was primarily predicted by heart rate, sweat rate, differences between *T*_core_ and *T*_skin_, wet‐bulb globe temperature, initial *T*_core_, running speed and maximal oxygen uptake, in this order of importance (β power values corresponded to 0.462, −0.395, 0.393, 0.327, 0.277, 0.244 and 0.228, respectively). In conclusion, several factors predict *T*_core_ in athletes subjected to self‐paced running under environmental heat stress. Moreover, considering the conditions investigated, heart rate and sweat rate, two practical (non‐invasive) variables, have the highest predictive power.

## Abstract (highlights)

- **What is the central question of this study?**
  The aim was to identify the factors predicting the body core temperature of athletes at the end of a 10 km self‐paced run in a hot environment.
- **What is the main finding and its importance?**
  Hyperthermia in athletes subjected to self‐paced running depends on several factors, highlighting the integrated control of core temperature during exercise under environmental heat stress. Five of the seven variables that significantly predicted core temperature are not invasive and, therefore, practical for use outside the laboratory environment: heart rate, sweat rate, wet‐bulb globe temperature, running speed and maximal oxygen consumption.

## 1 INTRODUCTION

Endurance running competitions are popular sporting events with increasing numbers of participants (Cushman et al., 2014; El Helou et al., 2012; Noakes, 2003). In these competitions, athletes experience significant thermoregulatory strain (Racinais et al., 2019) because they exercise at high intensities (associated with fast rates of metabolic heat production) for prolonged periods, often under environmental heat stress (Mündel, 2020; Vihma, 2010). Thus, under conditions of exercise‐heat stress, measuring body core temperature (*T*_core_) is paramount to determining the thermoregulatory strain of athletes (Eggenberger et al., 2018).

Increased *T*_core_, a hallmark physiological response to physical exercise, results from a transient imbalance between the rates of body heat production and heat loss (Webb, 1995). However, standard measurement procedures for *T*_core_ (e.g., rectal or oesophageal temperature probes) are not practical for extended use outside the laboratory environment (Eggenberger et al., 2018). Therefore, it is essential to identify variables that predict the exercise‐induced increase in *T*_core_, including valid and non‐invasive surrogate measures for monitoring thermoregulatory strain.

Although increased *T*_core_ favours metabolic responses during exercise (J. Barcroft, 1909; H. Barcroft & Edholm, 1943; Koga et al., 1997), marked hyperthermia is associated with reduced endurance (Cheung & Sleivert, 2004) and even the occurrence of heat illnesses (Armstrong et al., 2007). Regarding physical performance, *T*_core_ has direct and indirect effects that limit the ability to exercise for prolonged periods (Cheung & Sleivert, 2004). For example, increased *T*_core_, possibly brain temperature, changes EEG activity (Nybo & Nielsen, 2001b), increases perceived exertion (Nybo & Nielsen, 2001b) and reduces the central drive for muscle contractions (Moraes et al., 2019; Nybo & Nielsen, 2001a). Concerning the health of athletes, severe hyperthermia has been associated with impaired intestinal function (Pals et al., 1997; Pires et al., 2018), endotoxaemia (Gisolfi, 2000) and a higher incidence of heat stroke, the most severe heat illness (Armstrong et al., 2007). Thus, identification of factors that predict *T*_core_ will allow the development of more effective strategies to mitigate the degradation of endurance performance in the heat and minimize the incidence of exertional heat stroke, a life‐threatening illness (Bouchama et al., 2022).

The *T*_core_ level attained during prolonged aerobic exercise depends on the intensity of physical exertion; the higher the metabolic rate, the higher the steady‐state *T*_core_ (Nielsen, 1966; Saltin & Hermansen, 1966; Sawka et al., 2011). This relationship is independent of various environmental conditions, mainly when evaporative heat loss can compensate for augmented metabolic heat production (i.e., compensable heat stress; Sawka et al., 2011). However, when athletes exercise at moderate to vigorous intensities in extreme environmental conditions, their *T*_core_ will increase continuously, thereby not reaching a steady state (i.e., non‐compensable heat stress; Sawka et al., 2011).

Factors other than environmental conditions and exercise intensity can also influence running‐induced hyperthermia. For example, greater sweating capacity and enhanced evaporative heat loss resulted in cooler skin temperature (*T*_skin_) and attenuated exercise hyperthermia in heat‐acclimated individuals (Magalhães et al., 2010). Furthermore, augmented *T*_core_‐to‐*T*_skin_ differences allow for attaining greater *T*_core_ levels during exercise (Cheuvront et al., 2010), and a high maximal oxygen uptake (${\dot{V}}_{O_{2}\max}$) improves the tolerance to an elevated *T*_core_ at exhaustion (Selkirk & McLellan, 2001). Thermoregulatory responses are also influenced by body mass (Cramer & Jay, 2014) and percentage changes in body mass (Montain et al., 1995), which indicate hydration status. In addition, the heart rate (HR), which is mainly related to metabolic demands during exercise, is influenced by blood flow redistribution from the central to peripheral circulation to facilitate heat exchange on the body surface (González‐Alonso et al., 2008; Niedermann et al., 2014). Finally, although initial *T*_core_ does not seem to affect *T*_core_ attained at fatigue, at least in humans subjected to fixed‐intensity exercises (González‐Alonso et al., 1999; Nielsen et al., 1997), previous evidence in experimental animals revealed that initial *T*_core_ had a relevant predictive power (Andrade et al., 2023). Therefore, the role of this variable in predicting hyperthermia in exercising subjects merits further investigation.

The information mentioned earlier indicates that several factors might be associated with the level of hyperthermia during exercise. However, to our knowledge, no study has investigated the predictive ability of these factors during self‐paced exercise using multivariate analysis. Previous studies predicted *T*_core_ using data from fixed‐intensity exercise (Eggenberger et al., 2018; Havenith, 2001; Laxminarayan et al., 2018; Niedermann et al., 2014; Richmond et al., 2015) with limited ecological validity (Goulet, 2013). Moreover, performance in fixed‐intensity exercise to exhaustion is less reliable than during self‐paced exercise (Currell & Jeukendrup, 2008). In this sense, self‐paced exercise reproduces the demands of real‐world competitions (i.e., the work rate varies, resulting in transitions between intensity domains; Azevedo et al., 2021), and the performance of trained individuals in this exercise is highly reproducible (Mündel et al., 2023; Tyler & Sunderland, 2008). Considering these points, we investigated factors that could predict the level of *T*_core_ attained in trained individuals during self‐paced treadmill running.

The aim of the present study was to examine the *T*_core_ values attained at the end of a 10 km time trial (end‐*T*_core_) under environmental heat stress. Furthermore, the data were analysed using multiple linear regression to identify which factors predicted end‐*T*_core_. These multiple linear regression analyses included most anthropometric and physiological variables studied or only those variables considered practical (i.e., non‐invasive, unrelated to *T*_core_ measurement). Therefore, we hypothesized that marked hyperthermia would be reported in the present experimental conditions and that environmental heat stress, cardiovascular strain, cutaneous heat loss, average running speed and ${\dot{V}}_{O_{2}\max}$ would, collectively, predict the *T*_core_ value attained when a 10 km race is finished. Moreover, we expected that analysing only practical variables would also lead to robust predictive models, as previously reported (Eggenberger et al., 2018).

## 2 MATERIALS AND METHODS

### 2.1 Ethical approval

All experimental procedures were approved by the Human Research Ethics Committee of the Universidade Federal de Minas Gerais (protocols 80315917.9.0000.5149, 40854115.9.0000.5149, 97810818.9.0000.5149 and 55700416.6.0000.5149) and conformed to the ethical standards set by the 1964 *Declaration of Helsinki* and its later amendments, except for registration in a database. In addition, the participants signed an informed consent form and were aware that they could discontinue participation without presenting any justification.

### 2.2 Participants

The sample for the present study consisted of 46 individuals (40 males and 6 females) with the following characteristics (mean ± SD): age 32.4 ± 4.9 years, body mass 75.6 ± 3.3 kg, height 174.8 ± 2.0 cm, body fat 17.4 ± 1.4% and ${\dot{V}}_{O_{2}\max}$ 56.2 ± 7.2 ml kg^−1^ min^−1^). The individuals were engaged in endurance (i.e., running) training and were not specifically heat acclimated. For female participants, care was taken to avoid possible influences caused by different phases of the menstrual cycle (Cheung & Sleivert, 2004; Janse de Jonge, 2003; Minahan et al., 2017); only women who had been using low‐dose combined monophasic oral contraceptives regularly for ≥12 months were recruited. They were tested during the active phase of the contraceptive pill (from the 2nd to the 21st day) (Amorim et al., 2008; Mee et al., 2015; Minahan et al., 2017).

The data used in the present study were obtained from four studies conducted in our laboratory in which individuals were subjected to a 10 km time trial under environmental heat stress. The data set, hosted on a not‐for‐profit repository (https://doi.org/10.6084/m9.figshare.21508242 and https://doi.org/10.6084/m9.figshare.21508239), contains data from control subjects of these investigations. We ensured that the experimental design varied only slightly between the studies that provided data for the present analyses. The following sections describe the common methodological aspects of these studies.

### 2.3 Preliminary procedures

Body mass and height (MF‐100; Filizola, Brazil) were measured, and body fat percentage (Jackson & Pollock, 1978) was determined. Next, incremental intensity running tests were performed on the HPX‐350 treadmill (Total Health, Brazil) to determine ${\dot{V}}_{O_{2}\max}$ in a controlled environment [dry ambient temperature (*T*_dry_) = 23.6 ± 0.7°C and relative humidity = 53 ± 3%]. The test (*n* = 57) started at a speed of 6.7 km h^−1^ and an incline of 10%. Then, the speed and incline were increased by 1.3 km h^−1^ and 2%, respectively, every 3 min until the participants were fatigued. Respiratory variables were continuously registered breath by breath using a gas analyser (GasSys2 module; BIOPAC System, USA) calibrated before each test. Heart rate (RS800; Polar, Finland) and the rate of perceived exertion (6–20 RPE scale; Borg, 1982) were recorded at the end of each stage and at fatigue. During this incremental test, at least two of the following criteria were required to determine that ${\dot{V}}_{O_{2}\max}$ was achieved: (1) no increase in the O_2_ uptake or HR despite increased exercise intensity; (2) an RPE of >17 on the Borg scale; (3) a respiratory exchange ratio of >1.10; and (4) an HR of >90% of the age‐predicted maximum (i.e., 220 minus age). The ${\dot{V}}_{O_{2}\max}$ was considered to be the highest O_2_ uptake recorded during the test.

Owing to technical reasons, ${\dot{V}}_{O_{2}\max}$ was estimated (George et al., 2009) in a subset of our sample (*n* = 18 individuals). The participants exercised until they reached 70% of their age‐predicted maximal HR (HR_max_). Then, they went through three stages with progressive increases in exercise intensity from 4.8 to 6.4 km h^−1^ in stage 1, from 6.5 to 9.6 km h^−1^ in stage 2, and >9.6 km h^−1^ in stage 3. The self‐selected pace in each stage was determined by the participant, who informed the examiner to increase or decrease the treadmill speed until a comfortable pace was found (i.e., not too easy and not too hard). The self‐selected pace was set within the first 20 s of each stage. When participants reached ≥70% of their age‐predicted HR_max_ during a given stage, that stage was ended, and then the treadmill speed was decreased to a value corresponding to a slow walk for ∼2–5 min. The participants progressed to the next test stage if they did not reach 70% of their HR_max_. The selected treadmill speed, exercise HR and RPE were recorded during the last 15 s of each completed exercise stage. This protocol presented a correlation coefficient of 0.92 with the ${\dot{V}}_{O_{2}\;\max}$ values measured by spirometry (George et al., 2009).

All participants were asked to avoid performing strenuous physical activities and drinking alcoholic or caffeinated beverages for ≥24 h before the incremental testing, familiarization session and experimental trial. In addition, participants were instructed to record their 24 h dietary habits before the trial. When subjected to more than one trial, they were told to reproduce the same pattern before all subsequent exercise sessions.

### 2.4 Experimental design and procedures

Some participants were subjected to more than one experimental trial; therefore, the number of trials included (*n* = 75) was higher than the number of participants (*n* = 46). All participants underwent a familiarization session and at least one experimental trial. The familiarization and experimental sessions were separated by ≥7 days and initiated at the same time on different days (<1 h difference). Most trials (*n* = 64) were conducted in the morning, but a few (*n* = 11) occurred in the late afternoon/early evening.

All experimental sessions took place inside an environmental chamber (WMD 1150‐5; Russels Technical Products, USA) set to control the dry‐bulb temperature (*T*_dry_) at 33.0 ± 0.2°C. The *T*_dry_ and wet‐bulb temperature (*T*_wet_; 26.8 ± 2.2°C) were measured using a Thermal Stress Meter (TGD 400; Instrutherm, Brazil) and were used to calculate the relative humidity, which varied from 45 to 73% among experimental trials (61 ± 9%; mean ± SD).

Participants wore shorts, socks and appropriate running shoes. In the case of female participants, they also used sports bras. During the familiarization session and experimental trials, participants were allowed to drink water ad libitum. The temperature of the water ingested was 12.0 ± 4.4°C. Most likely, ingesting water at this temperature did not influence the *T*_core_ increase, as supported by a previous study showing that ad libitum water at 10°C did not affect heat storage rate and body temperatures during a 40 km self‐paced cycling trial in the heat (De Carvalho et al., 2015).

After an overnight fast, participants arrived at the laboratory 2 h before the experimental trial. Notably, in the experiments performed in the late afternoon/early evening, participants fasted for ≥3 h, with only water allowed. Upon arrival, each individual provided a urine sample to check their hydration status using a portable refractometer (Uridens; Inlab, Brazil). Next, they ate a standardized meal and drank 500 mL of water. Afterwards, the participants inserted a disposable rectal probe (MEAS 4400‐Series Temperature Probe; Measurement Specialties, USA) ∼11 cm past the anal sphincter for measurement of *T*_core_. In addition, an HR monitor was placed on the chest of each participant, and skin temperature measurements were taken from the chest, arm and thigh with an infrared thermometer (model 62 MAX; Fluke, USA). The *T*_core_, HR and skin temperatures were recorded immediately before exercise and at every 1 km.

Before the experimental trial, participants emptied their bladders and weighed naked, using only the rectal probe. They all started the experimental trial in a euhydrated state (urine specific gravity = 1.016 ± 0.008) (Armstrong et al., 1998). Then, participants entered an environmental chamber, where they rested in a chair for 10 min before recording pre‐exercise measurements (i.e., the 0 km time point). Next, an examiner initiated the treadmill, and participants could change the running speed at any time, thus characterizing the run as self‐paced exercise intensity. The treadmill incline was always maintained at 1%. Artificial wind (on average, 3.9 km h^−1^) was created by an electrical fan placed in front of the participants. While running, the individuals were blinded to the time elapsed and only received feedback on the total distance covered through the treadmill display. Upon completing the exercise, they left the environmental chamber, received a towel to dry their bodies, and then the postexercise body mass was recorded. Again, they were weighed naked, using only the rectal probe. After this procedure, the individuals were taken to a changing room, where they discarded the rectal probe.

### 2.5 Calculated variables

The values obtained at the three skin sites through infrared thermography were used to calculate the mean skin temperature according to the following equation (Roberts et al., 1977): *T*_skin_ = (*T*_chest_ × 0.43) + (*T*_arm_ × 0.25) + (*T*_thigh_ × 0.32). The indoor wet‐bulb globe temperature index (WBGT) was calculated according to the following equation (Lemke & Kjellstrom, 2012): WBGT = (0.7 × *T*_wet_) + (0.3 × *T*_dry_).

The *T*_core_‐to‐*T*_skin_ difference was calculated; this difference represents the heat exchange gradient between the body core and periphery and is possibly one of the mechanisms by which hot skin impairs endurance in the heat (Sawka et al., 2012). Whole‐body sweating was calculated as the difference between the pre‐ and postexercise body masses, adding the mass of water ingested. Then, whole‐body sweating was divided by the time taken to complete the 10 km run to obtain the sweat rate (in litres per hour) (Dugas et al., 2009). It is worth mentioning that a dry towel was used to remove the sweat from the skin surface of participants before measuring postexercise body mass. Also, the postexercise urine collection was done after the participants had been weighed.

The percentage change in body mass (%ΔBM) was calculated as the difference between pre‐ and postexercise body mass, adding the mass of water ingested; the resulting value was divided by pre‐exercise body mass, then multiplied by 100.

### 2.6 Statistical analysis

Data normality and homoscedasticity were assessed using the Shapiro–Wilk and Levene tests, respectively. Both tests revealed no significant effects; therefore, the data were expressed as the mean ± SD.

Initially, we described the mean value, SD, maximum and minimum values and the coefficient of variation (CV; i.e., data dispersion around the mean value of the sample) of the following variables: *T*_core_ at the 10th kilometre, pre‐exercise *T*_core_, mean *T*_skin_, *T*_core_‐to‐*T*_skin_ difference, sweat rate, water intake, average running speed, ${\dot{V}}_{O_{2}\max}$, HR, pre‐exercise body mass, ∆BM%, *T*_dry_, *T*_wet_, absolute and relative humidity and WBGT. To characterize the 10 km time trial further, *T*_core_, mean *T*_skin_, *T*_core_‐to‐*T*_skin_ difference, average speed and HR were compared between running distances using one‐way repeated‐measures ANOVAs. For post hoc pairwise comparisons, Tukey's test or Student's paired *t*‐test was performed according to the CV of the variable analysed (Huberty & Morris, 1988).

To identify which variables might predict the end‐*T*_core_, we performed multiple linear regression analyses using the following independent variables: WBGT, average speed, initial *T*_core_, body mass, *T*_core_‐to‐*T*_skin_ difference (or mean *T*_skin_), sweat rate, ${\dot{V}}_{O_{2}\max}$, HR and %∆BM. The first and last authors selected these variables based on the theoretical background highlighted in the Introduction. Initially, we ran a force‐entry multiple linear regression analysis to check whether multicollinearity had occurred between variables. Then, hierarchical multiple linear regression analyses were conducted because, as mentioned earlier, independent variables were selected based on researcher expertise (Jeong & Jung, 2016). Information related to the hierarchical regression model [adjusted *R* ^2^, standard error of the estimate (SEE), change statistics and collinearity statistics] and the regression coefficients (unstandardized and β weights) are reported in the Results section. It is noteworthy that the unstandardized regression coefficients cannot be used to establish the relative importance of specific variables within a regression equation because they are based on different units of measurement (Portney & Watkins, 2015). Therefore, the standardized regression coefficients (i.e., converted to *z*‐scores), called β weights, are presented to provide the reader with a complete and practical interpretation of the observed relationships (Portney & Watkins, 2015).

The graphs were created using the software SigmaPlot (v.11.0; Systat Software, USA), and the statistical analyses were performed using the software IBM SPSS (v.19.0; IBM Corporation, USA). In all analyses, the significance level was set at *P* < 0.05.

## 3 RESULTS

The average running speed was 11.2 ± 1.6 km h^−1^, with the slowest and fastest speeds corresponding to 8.0 and 14.9 km h^−1^, respectively. The initial *T*_core_ ranged from 35.7 to 37.6°C, with average values of 36.8 ± 0.4°C. The end‐*T*_core_ corresponded to 39.6 ± 0.5°C, with 38.3 and 40.8°C being the minimum and maximum temperature values attained, respectively. On average, *T*_core_ increased by 2.7°C during the 10 km trial (Table 1).

**TABLE 1:** Mean values, SDs, coefficients of variation, minimum and maximum values of the variables measured in the individuals subjected to the 10 km time trial under environmental heat stress.

Variable	Mean	SD	CV (%)	Minimum	Maximum
*T*_core_ at the 10th kilometre (°C)	39.6	0.5	1.3	38.3	40.8
Initial *T*_core_ (°C)	36.8	0.4	1.0	35.7	37.6
Mean *T*_skin_ (°C)	33.9	2.1	6.3	28.9	36.3
*T*_core_‐to‐*T*_skin_ difference (°C)	4.3	2.2	50.9	2.0	9.3
Sweat rate (L h^−1^)	2.15	1.04	48.5	0.95	5.58
Water intake (mL)	266	222	83.5	0	860
Running speed (km h^−1^)	11.2	1.6	14.0	8.0	14.9
${\dot{V}}_{O_{2}\max}$ (mL kg^−1^ min^−1^)	56.2	7.2	12.9	41.5	70.1
Heart rate (beats min^−1^)	156	11	6.9	132	178
Pre‐exercise body mass (kg)	77.3	11.1	14.3	50.4	102.0
∆BM (%)	1.9	0.5	26.1	0.9	3.6
WBGT (°C)	28.7	1.5	5.3	25.1	31.0
Dry temperature (°C)	33.0	0.2	0.5	32.7	33.5
Wet temperature (°C)	26.8	2.2	8.1	21.7	30.0
Absolute humidity (g m^−3^)	21.9	3.0	13.6	16.0	27.0
Relative humidity (%)	61	9	14.0	45	73

*Note*: For *T*_skin_, *T*_core_‐to‐*T*_skin_ difference, heart rate, WBGT, dry and wet temperatures, absolute and relative humidity, we first calculated the mean value of the data collected during the 10 km run for each individual.

Abbreviations: %∆BM, percentage change in body mass; CV, coefficient of variation; *T*_core_ = body core temperature; *T*_skin_, mean skin temperature; ${\dot{V}}_{O_{2}\max}$, maximal oxygen uptake; WBGT, wet‐bulb globe temperature.

The variable with the lowest CV was *T*_dry_, deliberately controlled at 33°C in all experimental trials. The variables with the lowest CV following *T*_dry_ were *T*_core_ values measured at the beginning and end of the time trial (∼1%). In contrast, the variables with the highest CV (ranging from 48.5 to 83.5%) were water intake, sweat rate and *T*_core_‐to‐*T*_skin_ difference (Table 1).

**FIGURE 1:**
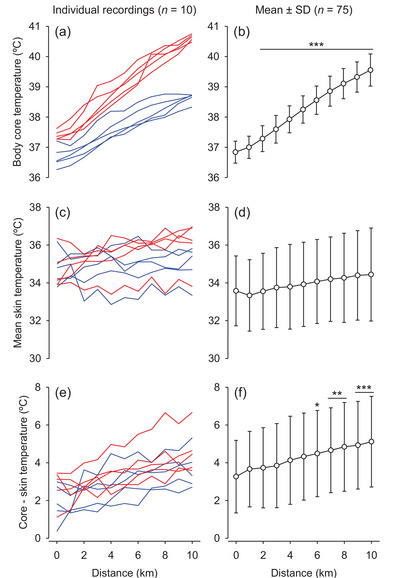
Body core temperature (*T*_core_; a,b), mean skin temperature (c,d) and *T*_core_ minus skin temperature (e,f) during the 10 km time trial under environmental heat stress. The panels on the left show 10 individual recordings: five from subjects with the highest end‐*T*_core_ (red lines) and the other five from subjects with the lowest end‐*T*_core_ (blue lines). The panels on the right show the mean and SD values of data extracted from the 75 recordings analysed. ^*^ *P* < 0.05, ^**^ *P* < 0.01 and ^***^ *P* < 0.001 compared with 0 km (i.e., pre‐exercise).

The *T*_core_ increased at the second kilometre compared with pre‐exercise and continued to increase gradually throughout the 10 km time trial (*F* = 323.199; *P* < 0.001; Figure 1a,b). The continuous increase in *T*_core_ indicates that the experimental conditions (i.e., exercise intensity plus environmental conditions) were characterized as non‐compensable heat stress. In addition, *T*_skin_ was modified over the running protocol (*F* = 2.112; *P* = 0.021; Figure 1c,d); however, the post hoc test failed to reveal differences in pairwise comparisons (*P* > 0.05). Finally, the *T*_core_‐to‐*T*_skin_ difference was higher from the sixth kilometre than at pre‐exercise (*F* = 5.325; *P* < 0.001; Figure 1e,f).

**FIGURE 2:**
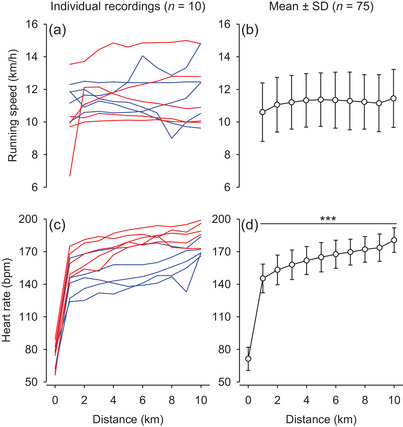
Running speed (a,b) and heart rate (c,d) during the 10 km time trial under environmental heat stress. The panels on the left show 10 individual recordings: five from subjects with the highest end‐core temperature (*T*_core_; red lines) and the other five from subjects with the lowest end‐*T*_core_ (blue lines). The panels on the right show the mean and SD values of data extracted from the 75 recordings analysed. ^***^ *P* < 0.001 compared with 0 km (i.e., pre‐exercise).

The running speed of participants did not vary significantly over the 10 km time trial (*F* = 1.469; *P* = 0.155; Figure 2a), although relevant inter‐individual differences in running strategy existed (Figure 2b). Similar to *T*_core_, HR increased at the first kilometre compared with pre‐exercise and continued to increase gradually throughout the exercise session (*F* = 421.395; *P* < 0.001; Figure 2c,d).

The RPE and HR were assessed to determine how the participants experienced physical exertion when completing the 10 km run as fast as possible. At the end of the exercise, the RPE and HR of the participants were 18 ± 2 and 181 ± 11 beats min^−1^, respectively. This average HR corresponds to 96% of the age‐predicted HR_max_.

We performed multiple linear regression analyses to understand the predictors of *T*_core_ attained by participants at the end of the time trial under environmental heat stress. Initially, we included 10 variables (i.e., WBGT, running speed, initial *T*_core_, body mass, *T*_core_‐to‐*T*_skin_ difference, mean *T*_skin_, sweat rate, ${\dot{V}}_{O_{2}\max}$, HR and ∆BM%) in a forced‐entry regression model. This analysis led to an adjusted *R* ^2^ = 0.876 and SEE = 0.187. However, multicollinearity was observed between the mean *T*_skin_ and *T*_core_‐to‐*T*_skin_ difference [tolerance < 0.10 (0.09 for both variables) and variance inflation factor > 10 (107.2 and 106.6, respectively)]. Because multicollinearity hampered data interpretation, we conducted two additional regression analyses with nine independent variables: one analysis including the *T*_core_‐to‐*T*_skin_ difference (shown below) and the other including the mean *T*_skin_ (with lower adjusted *R* ^2^ and, therefore, shown in the supplementary material; https://doi.org/10.6084/m9.figshare.21508236).

The regression with nine independent variables (WBGT, running speed, initial *T*_core_, body mass, *T*_core_‐to‐*T*_skin_ difference, sweat rate, ${\dot{V}}_{O_{2}\max}$, HR and %∆BM) led to an adjusted *R* ^2^ = 0.553. However, the change statistics showed that pre‐exercise body mass (*F* change = 0.134; *P* = 0.715), *T*_core_‐to‐*T*_skin_ difference (*F* change = 0.063; *P* = 0.803), and ${\dot{V}}_{O_{2}\max}$ (*F* change = 0.510; *P* = 0.478) did not contribute significantly to the hierarchical regression model. Therefore, we tried to improve the robustness of our model (i.e., to increase adjusted *R* ^2^ and reduce SEE) by removing these three variables, one at a time; reducing the number of independent variables was necessary owing to the number of recordings available (*n* = 75). Removing body mass generated the most robust statistical model with eight independent variables (Table 2). This analysis led to the following regression equation, with an adjusted *R* ^2^ = 0.560 and SEE = 0.352:

**TABLE 2:** Summary of the multiple linear regression analysis using most anthropometric and physiological variables available and those analyses using only practical variables.

Independent variable	Adjusted *R* ^2^	SEE	*F* change	*F* change significance	β weight
Model with most anthropometric and physiological variables available					
WBGT	0.159	0.487	15.01	<0.001	0.327
Running speed	0.174	0.482	2.31	0.133	0.244
Initial *T*_core_	0.335	0.433	18.48	<0.001	0.277
*T*_core_‐to‐*T*_skin_ difference	0.326	0.436	0.03	0.862	0.363
Sweat rate	0.360	0.425	4.68	0.034	‐0.395
${\dot{V}}_{O_{2}\max}$	0.355	0.426	0.50	0.483	0.228
HR	0.554	0.355	31.26	<0.001	0.462
%ΔBM	0.560	0.352	1.98	0.164	0.122
Model with five practical variables					
HR	0.285	0.449	30.43	<0.001	0.548
Sweat rate	0.301	0.444	2.74	0.102	‐0.030
WBGT	0.398	0.412	12.61	0.001	0.304
Running speed	0.411	0.407	2.59	0.112	0.023
${\dot{V}}_{O_{2}\max}$	0.435	0.399	3.89	0.053	0.232
Model with two practical variables (Eggenberger et al., 2018)					
HR	0.285	0.449	30.43	<0.001	0.533
*T*_chest_	0.309	0.441	3.57	0.063	0.183

Abbreviations: %ΔBM, percentage change in body mass; HR, heart rate; SEE, standard error of the estimate; *T*_chest_, skin temperature measured on the chest surface; *T*_core_, body core temperature; *T*_skin_, mean skin temperature; ${\dot{V}}_{O_{2}\max}$, maximal oxygen consumption; WBGT, wet‐bulb globe temperature.

*T*_core_ at the 10th kilometre = 15.768 + (0.115 × WBGT) + (0.082 × running speed) + (0.404 × initial *T*_core_) + (0.089 × *T*_core_‐to‐*T*_skin_ difference) − (0.201 × sweat rate) + (0.017 × ${\dot{V}}_{O_{2}\max}$) + (0.023 × HR) + (0.130 × %∆BM)

**FIGURE 3:**
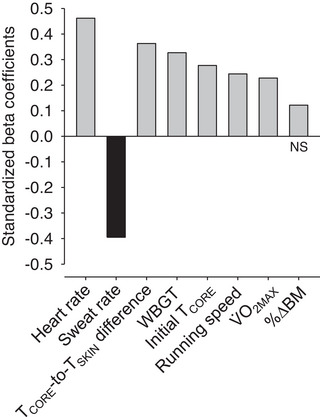
Standardized β coefficients of the eight independent variables included in the multiple linear regression analysis regarding the body core temperature (*T*_core_) attained at the end of the 10 km time trial under environmental heat stress. The grey bars indicate positive coefficients (direct influences), whereas the black bar indicates a negative coefficient (inverse influence). Notably, the variables with greater coefficients are the most important for predicting *T*_core_ at the 10th kilometre. Abbreviations: %ΔBM, percentage change in body mass; NS, not significantly different from zero; *T*_skin_, mean skin temperature; ${\dot{V}}_{O_{2}\max}$, maximal oxygen consumption.

Standardized β coefficients corresponded to 0.462, −0.395, 0.363, 0.327, 0.277, 0.244, 0.228 and 0.122 for HR, sweat rate, differences between *T*_core_ and *T*_skin_, WBGT, initial *T*_core_, running speed, ${\dot{V}}_{O_{2}\max}$ and %∆BM, respectively (Figure 3; Table 2). These data indicate that HR and %∆BM were, respectively, the variables with the best and worst predictive power used in the model. Moreover, these β values mean, for example, that *T*_core_ at 10 km increases by 0.462 (in SDs) when the HR increases by one SD, assuming other variables in the model are held constant.

We also determined whether the β coefficients of the eight independent variables mentioned earlier were different from zero, which was the case for the following variables: WBGT (*t* = 3.389; *P* = 0.001), running speed (*t* = 2.178; *P* = 0.033), initial *T*_core_ (*t* = 3.052; *P* = 0.003), *T*_core_‐to‐*T*_skin_ difference (*t* = 2.557; *P* = 0.013), sweat rate (*t* = −2.812; *P* = 0.006), ${\dot{V}}_{O_{2}\max}$ (*t* = 2.163; *P* = 0.034) and HR (*t* = 5.463; *P* < 0.001). In contrast, %∆BM (*t* = 1.408; *P* = 0.164) was not significantly different from zero. Interestingly, the inclusion of %ΔBM still increased the adjusted *R* ^2^ and reduced SEE; therefore, we decided to keep this variable in the final model.

It is noteworthy that models with seven independent variables (by excluding either *T*_core_‐to‐*T*_skin_ difference or ${\dot{V}}_{O_{2}\max}$ alongside pre‐exercise body mass) were not more robust than the model with eight variables described earlier.

In the analysis with eight independent variables, 11 of the 75 recordings were made in the late afternoon/early evening. Therefore, we found it essential to exclude these 11 recordings and thus verify whether they had influenced our findings. The adjusted *R* ^2^ was relatively similar between the analyses that included all recordings and only the recordings in the morning (0.560 vs. 0.543). Notwithstanding, the adjusted *R* ^2^ was also relatively similar between the analyses that included all recordings and the 69 recordings obtained in men (0.560 vs. 0.574), thus suggesting that biological sex was not a confounding factor in our analysis.

Finally, we ran two regression analyses with only non‐invasive variables to predict end‐*T*_core_ (Table 2). The first analysis included five practical variables of the seven independent variables that reached statistical significance in our final model described in Figure 3. The practical variables were unrelated to *T*_core_ measurement and were as follows: HR, sweat rate, WBGT, running speed and ${\dot{V}}_{O_{2}\max}$. This analysis had an adjusted *R* ^2^ = 0.435 and SEE = 0.399 and revealed that the β coefficients of HR (*t* = 5.966; *P* < 0.001) and WBGT (*t* = 2.986; *P* = 0.004) were significantly different from zero, whereas the coefficient of ${\dot{V}}_{O_{2}\max}$ was close to reaching statistical significance (*t* = 1.971; *P* = 0.053).

The second analysis included the two practical variables identified as significant predictors by Eggenberger et al. (2018) and had an adjusted *R* ^2^ = 0.309 and SEE = 0.441. This analysis also showed that the β coefficient of HR was significantly different from zero (*t* = 5.510; *P* < 0.001), whereas the coefficient of *T*_chest_ (*t* = 1.890; *P* = 0.063) was close to reaching statistical significance.

Standardized β coefficients and additional information regarding the two analyses with practical variables are presented in Table 2.

## 4 DISCUSSION

The data extracted from 75 individual recordings were assessed using multiple linear regression analysis to identify which factors predict the *T*_core_ at the end of the 10 km time trial. Our main finding was that HR, sweat rate, *T*_core_‐to‐*T*_skin_ difference, WBGT, initial *T*_core_, running speed and ${\dot{V}}_{O_{2}\max}$ were, in this order of importance, the factors predicting end‐*T*_core_. As a secondary finding, the analyses conducted with only practical variables could significantly predict end‐*T*_core_. However, their predictive power was less robust than the analysis including non‐practical variables (i.e., *T*_core_‐to‐*T*_skin_ difference and initial *T*_core_). These findings are particularly relevant considering the accelerated global warming in recent decades, the increased incidence of heat waves (Dosio et al., 2018; Kenney et al., 2022), and several sports competitions held in hot and humid environments (Mündel, 2020).

Heart rate was the variable that best predicted end‐*T*_core_, and this finding supports previous data (Eggenberger et al., 2018; Laxminarayan et al., 2018). During prolonged aerobic exercise under environmental heat stress, the demands of an elevated *T*_core_ for increasing skin blood flow compete with the metabolic demands to increase blood flow to active muscles, thus generating accentuated increases in HR and cardiac output (González‐Alonso et al., 2008). Augmented HR also plays an essential role in maintaining blood pressure when individuals are dehydrated by sweating and when blood flow is redirected from the central circulation to the skin circulation to dissipate body heat (Niedermann et al., 2014). Therefore, the increase in HR is a response elicited by reduced plasma volume and blood flow redistribution owing to the activation of heat‐defense thermoeffectors (i.e., sweating and skin vasodilatation). In this regard, it should be noted that %ΔBM, an indirect dehydration index (Sawka et al., 2007), was a variable with low predictive power, which aligns with previous observations (Richmond et al., 2015).

Although the participants exhibited an average sweat rate of 2.2 L h^−1^, *T*_core_ increased continuously throughout the trial, indicating that our experimental conditions (physical exertion intensity plus environmental conditions) can be characterized as non‐compensable heat stress (Sawka et al., 2011). The *T*_core_ increased by 2.7°C on average over 53.9 min, corresponding to a heating rate of ∼0.05°C min^−1^. As expected, the multiple linear regression analysis indicated that sweat rate was inversely related to end‐*T*_core_, showing that the participants who sweated more had attenuated hyperthermia. The sweat rate does not necessarily indicate the evaporative heat loss, particularly considering that both the absolute and relative humidity had a coefficient of variation of ∼14%. Evaporative heat loss is expected to be more effective in dry conditions, thereby attenuating the exercise‐induced increase in *T*_core_, as previously reported (Che Muhamed et al., 2016; Maughan et al., 2012). Regardless, increased sweat rate is consistently reported in heat‐acclimated subjects and contributes to their improved evaporative heat loss and attenuated exercise hyperthermia (Magalhães et al., 2010).

The *T*_core_‐to‐*T*_skin_ difference increased from the sixth kilometre compared with the pre‐exercise value. More importantly, this difference, which represents the heat exchange gradient between the deeper regions of the body and periphery (Sawka et al., 2012), significantly predicted end‐*T*_core_. Cheuvront et al. (2010) provided evidence that a greater *T*_core_‐to‐*T*_skin_ difference reduces the required skin blood flow to allow effective heat exchange between the body and the environment, thus preserving more blood volume in the central circulation (e.g., skeletal muscles, heart muscle and brain). Ensuring adequate blood perfusion to skeletal muscles improves aerobic exercise tolerance and allows the attainment of higher *T*_core_ (Cheuvront et al., 2010). Therefore, our results further support the importance of this thermoregulatory parameter in predicting the *T*_core_ level attained during aerobic exercise.

The environmental heat stress also predicted end‐*T*_core_, with higher WBGT values inducing more significant hyperthermia. Furthermore, the average WBGT values in our study corresponded to conditions associated with a high risk of hyperthermia and, possibly, heat illness for a typical marathon racer (Gonzalez, 1995). In the present study, the differences in WBGT were caused by distinct *T*_wet_ values (ranging from 21.7 to 30.0°C), with *T*_dry_ (ranging from 32 to 34°C) making a less significant contribution. This finding confirms previous experiments reporting that high levels of relative (Che Muhamed et al., 2016; Hayes et al., 2014; Teunissen et al., 2022) or absolute (Jenkins et al., 2023) humidity enhance whole‐body hyperthermia. During exercise‐heat stress, the limited thermal gradient between the skin and the environment restricts dry heat exchange, and cutaneous heat loss depends mainly on evaporation. However, sweating efficiency (i.e., the ratio between evaporated and secreted sweat) is determined primarily by air humidity (Alber‐Wallerström & Holmér, 1985); thus, exercising in hot and humid environments increases sweat drippage and decreases sweat vaporization, reducing evaporative heat loss and favouring the occurrence of severe hyperthermia.

Recent studies highlighted that evaporation from exposed and fully wetted skin depends mainly on the absolute humidity of the air (Jenkins et al., 2023; Lei et al., 2020). Therefore, we replaced the indoor WBGT with absolute humidity in the regression analyses. As a result, the adjusted *R* ^2^ slightly increased in the analysis including absolute humidity compared with that including WBGT (0.586 vs. 0.560). Despite this relevant finding, we decided to maintain the original analysis because, from a list of 61 thermal stress indicators assessed, the indoor WBGT was among those with the highest potential to quantify the physiological strain experienced by individuals working in the heat (Ioannou et al., 2022). Using the WBGT data, a widely used thermal stress indicator, also avoids the false impression that only absolute humidity, but not ambient temperature, determines *T*_core_ during exercise (i.e., *T*_dry_ was deliberately controlled in the present experiments). Moreover, the environmental factors analysed were restricted to *T*_dry_ and *T*_wet_, and future studies should also investigate solar or artificial radiation, in addition to wind speed and direction.

Initial *T*_core_ also had relevant predictive power. Specifically, the runners starting exercise with higher *T*_core_ values were the ones who finished it with more significant hyperthermia, and this finding either supports (Zheng et al., 2022) or does not support data obtained from previous studies (González‐Alonso et al., 1999; Hobson et al., 2009; Nielsen et al., 1997). In fixed‐intensity exercises to exhaustion (i.e., open‐ended exercises used in most of these earlier studies), it is unclear when physical exertion will be terminated. Therefore, when a high *T*_core_ level is attained, voluntary muscle activation is reduced, and RPE increases (Nybo & Nielsen, 2001a, 2001b, 2001a, 2001b), forcing the individuals to stop exercising. In contrast, in self‐paced exercise, the individuals have a task to accomplish and might override the inhibitory signals arising from elevated *T*_core_ values, thus keeping themselves motivated to complete the predetermined distance. The fact that performance in self‐paced exercise depends on motivation rather than an elevated *T*_core_ might have allowed the observation of the predictive role played by initial *T*_core_.

On average, the running speed of the participants did not vary significantly over the 10 km under environmental heat stress; this observation means that the participants could not sprint in the last kilometre. The final sprint, commonly observed in time trials, suggests that athletes exercise with a reserve capacity (Swart et al., 2009); however, under environmental heat stress, these individuals clearly adjust their pacing strategy and generate less power (Roelands et al., 2013). Most likely, our participants have already depleted their reserve capacity and/or motivation to cover the last kilometre at a fast pace, thus confirming previous findings involving time trials in the heat (De Carvalho et al., 2015; Maia‐Lima et al., 2017). More importantly, running speed also predicted the variance in end‐*T*_core_; therefore, the individuals running at faster speeds had more accentuated hyperthermia than those running at slower speeds. This result confirms the role played by exercise intensity in increasing *T*_core_ of athletes/trained individuals (Mohr et al., 2012; Pugh et al., 1967; Racinais et al., 2019; Zheng et al., 2022).

The ${\dot{V}}_{O_{2}\max}$ contributed to predicting end‐*T*_core_, in that the participants with higher ${\dot{V}}_{O_{2}\max}$ values achieved higher *T*_core_ than those with lower ${\dot{V}}_{O_{2}\max}$ values. This finding supports previous data (Cheung & McLellan, 1998; Selkirk & McLellan, 2001). Indeed, Selkirk & Mclellan (2001) reported that the main benefit of a high ${\dot{V}}_{O_{2}\max}$ was the ability to tolerate a higher *T*_core_ at exhaustion. On the contrary, individuals with lower ${\dot{V}}_{O_{2}\max}$ might experience greater circulatory strain at any given *T*_core_ during exercise‐heat stress owing to reduced blood and stroke volumes, and these responses might account for reducing the *T*_core_ tolerated at exhaustion (Hopper et al., 1988).

Multiple linear regression analyses, including most anthropometric and physiological variables studied or only the most practical ones (e.g., HR and *T*_chest_), were run to predict end‐*T*_core_. The analysis with several variables provided a more robust explanation (adjusted *R* ^2^ = 0.560) than the analyses with practical variables (adjusted *R* ^2^ = 0.435 and 0.309). Despite the method used, *R* ^2^ values of the present analyses are considerably lower than those of previous studies using several (Eggenberger et al., 2018; Niedermann et al., 2014; Richmond et al., 2015) or only practical variables (Eggenberger et al., 2018; Xu et al., 2013). These studies showed *R* ^2^ values ranging from 0.677 to 0.750. The contradiction between our findings and those from previous studies might be attributable to the different exercise protocols investigated: a self‐regulated exercise in our study versus fixed‐intensity exercise in the others. Investigation of the factors predicting the level of hyperthermia attained during self‐regulated exercise, which is more representative of real‐world competitions (Goulet, 2013), is a relevant advance provided by our study. In addition, the practical models, which also included the variable with the highest predictive power reported (i.e., HR), had a lower adjusted *R* ^2^ than the complete model containing eight variables. This finding highlights the integrated nature of *T*_core_ control during exercise.

We recently reviewed studies that measured *T*_core_ at fatigue and exhaustion in rats subjected to treadmill running. This systematic review revealed that *T*_core_ values attained at fatigue/exhaustion during incremental‐ and constant‐speed exercises were dependent mainly on ambient temperature, initial *T*_core_ and tolerance of physical exertion (Andrade et al., 2023); these outcomes are somewhat similar to those of the present study. Despite the different protocols used in the present study (self‐paced exercise) and the studies with rats (open‐ended exercise), the comparable findings suggest that somewhat similar mechanisms are implicated in body temperature regulation during exercise in the two species and that global warming and the higher incidence of heat waves (Dosio et al., 2018) might affect the performance and health of several animals.

The present study has certain limitations. First, we experienced technical problems with the gas analyser; therefore, a subset of ${\dot{V}}_{O_{2}\max}$ values was estimated, which might have added imprecision to our analysis. Nevertheless, the method used for estimation has a strong association with direct ${\dot{V}}_{O_{2}\max}$ measurements. A second limitation was that the O_2_ uptake by participants was not measured during time trials; this physiological variable can be used to calculate running economy, which is closely associated with metabolic heat production and might contribute to our understanding of the unexplained variance of our regression model. Moreover, perceptual variables were not included in the predictive model, and there is room for developing even more robust predictive models by considering perceived exertion and thermal perception as independent variables. Finally, only six recordings were obtained in women, thus preventing us from including biological sex as an independent variable in the multiple regression analysis. Future studies should include a higher proportion of women, thus helping to overcome their underrepresentation in research on thermoregulation in exercise (Hutchins et al., 2021). Moreover, the knowledge about the effects of oral contraceptive pill use in women athletes is still incipient. For example, Lei et al. (2019) showed small differences in thermoregulation even with contraceptive pill usage (i.e., *T*_core_ was 0.15°C higher in the quasi‐luteal than quasi‐follicular phase), possibly caused by a residual endogenous rhythm.

In conclusion, the present findings reveal that the level of hyperthermia (39.6 ± 0.5°C) in athletes subjected to a 10 km self‐paced run under environmental heat stress is predicted by several factors, thus highlighting the integrated control of *T*_core_ during exercise. In descending order of importance, the predicting factors were the HR, sweat rate, *T*_core_‐to‐*T*_skin_ difference, WBGT, initial *T*_core_, running speed and ${\dot{V}}_{O_{2}\max}$. Five of the seven variables that significantly predicted *T*_core_ are not invasive and are, therefore, practical for use outside the laboratory environment. However, the predictive power of these practical models was less robust than the model including non‐practical variables (i.e., *T*_core_‐to‐*T*_skin_ difference and initial *T*_core_). Lastly, our findings advance the knowledge on *T*_core_ prediction in recreational athletes during self‐paced exercise but indicate that there is still room to improve these predictive models, especially the ones including only the practical variables.

## AUTHOR CONTRIBUTIONS

Conception or design of the work: Marcelo Teixeira Andrade and Samuel Penna Wanner. Acquisition, analysis or interpretation of data for the work: Marcelo Teixeira Andrade, Matheus Mascarenhas Sacchetto Nunes‐Leite, Rúbio Sabino Bruzzi, Carlos Henrique Souza, João Paulo Uendeles‐Pinto and Samuel Penna Wanner. Drafting of the work or revising it critically for important intellectual content: Marcelo Teixeira Andrade, Luciano Sales Prado, Danusa Dias Soares, Dawit Albieiro Pinheiro Gonçalves, Cândido Celso Coimbra and Samuel Penna Wanner. All authors approved the final version of the manuscript and agree to be accountable for all aspects of the work in ensuring that questions related to the accuracy or integrity of any part of the work are appropriately investigated and resolved. All persons designated as authors qualify for authorship, and all those who qualify for authorship are listed.

## CONFLICT OF INTEREST

None declared.

## Supporting information


Statistical Summary Document

## Data Availability

The data used in the present manuscript are hosted on a not‐for‐profit repository (https://doi.org/10.6084/m9.figshare.21508242 and https://doi.org/10.6084/m9.figshare.21508239).
